# The Liquor Interests Denounced from the Scaffold

**Published:** 1913-06

**Authors:** 


THE LIQUOR INTERESTS DENOUNCED FROM THE SCAFFOLD.—Henry Brock, for murdering his paramour, was hanged at Austin, May 30th. He left a written statement, in which he arraigns the State of Texas for pursuing a policy that makes murderers and lunatics and ruins homes—the licensing of saloons. He said that he was the finished product of the saloon, had lived in that element all his life, and that his life was moulded (and ruined) by the environment. He was the ward boss and was feared and obeyed by all. He says that he procured (and distributed to those not entitled to vote) poll tax receipts and filled them out, and at election saw that the holders voted for the present administration—liquor. He says that he did this (and all other bidding) at the command of "certain interests" (local and not hard to guess) and rebukes them for deserting him. It is a powerful prohibition document. I have room to quote only one sentence: "I appeal to the young men and the young women of Texas to listen to the beckoning call and the earnest pleas of that noble class of womanhood and manhood, who stand for a higher order of things; they are the ones who ultimately will eliminate the environments which cause poor, weak human beings to fall a slave to sin and ruin."

Dr. Smith "Appreciated," that is, Dr. M. M., Dr. Matthew M., there is really but one Smith, but we have to differentiate. Once upon a time, before the Campbell Legislature gummed it up and gave us the anomaly called the State Board of Medical Examiners, a mixture of all sorts, we had a State Board of Medical Examiners composed of the flower of the regular profession. Dr. Sam R. Burroughs was president, and *the* Smith, M. M., was secretary. His efficiency as such has not been forgotten even at this late day. During the recent meeting of the State Medical Association at San Antonio, some of the former members of the board got together and gave Dr. Smith a luncheon at the St. Anthony, and sprung a pleasing surprise upon him—took his breath away, nearly. Dr. Frank Paschal produced from under the table or elsewhere a beautiful gold-headed cane, and in a brief but touching address, in which he recounted the services of Dr. Smith as secretary of the old board, presented it to him, assuring him that the survivors of the board bore it and him in grateful remembrance. Dr. J. H. Evans, another member, followed Dr. Paschal in a very pretty presentation speech. Dr. Smith, of course, was much affected and thrown off his guard, but nevertheless he responded in a pleasing and graceful manner, most appropriately. The writer had the honor to be the guest of the occasion.

